# Rethinking the senses and their interactions: the case for sensory pluralism

**DOI:** 10.3389/fpsyg.2014.01426

**Published:** 2014-12-10

**Authors:** Matthew Fulkerson

**Affiliations:** Department of Philosophy, University of CaliforniaSan Diego, La Jolla, CA, USA

**Keywords:** perception, multisensory processing, sense organs, pluralism in modeling, thermoreception

## Abstract

I argue for sensory pluralism. This is the view that there are many forms of sensory interaction and unity, and no single category that classifies them all. In other words, sensory interactions do not form a single natural kind. This view suggests that how we classify sensory systems (and the experiences they generate) partly depends on our explanatory purposes. I begin with a detailed discussion of the issue as it arises for our understanding of thermal perception, followed by a general account and defense of sensory pluralism.

## 1. Introduction

Start with two seemingly true statements: (i) We have many senses; (ii) They often interact.

These statements are now widely acknowledged and incorporated into recent work on perception, but they are also in deep tension with one another. Once we allow that the sensory modalities interact, and do so pervasively at multiple levels of sensory processing, with effects at all levels of our psychology (subpersonal, behavioral, and phenomenal), then it becomes difficult to make sense of what, exactly, these individual senses might be. Vision is less a single coherent modality than a complex collection of interacting subsystems. And that collection has features different in kind from those found in the auditory, vestibular, and nociceptive systems (of course, there are many similarities too). Indeed, it can become difficult to maintain the idea that we can have anything like a unified conception of sensory modalities and their interactions.

I start with a detailed discussion of human thermoreception, using it as a case study for the sort of tensions I describe above. I then discuss the general implications of this example, and propose a robust theoretical framework for addressing this tension. My claim is that we should abandon any single theoretical account of sensory interaction, and adopt a view according to which sensory systems and their interactions are classified in part by our explanatory purposes. The upshot of this proposal is that it allows us to fully acknowledge the deep interactions between sensory subsystems without thereby giving up entirely on the very idea of separate sensory modalities. The main target of my view is any form of sensory monism that assumes there will be a single, authoritative, and context free account of what it is to be a sensory modality and for an interaction between them to be “multisensory” or “multimodal.” On such a monist view, there should be a single determinate answer to the question of whether vestibular awareness or pain or any other putative sense counts as a sensory modality. I believe such a view is implausible and deeply problematic, and in what follows I offer a substantive alternative account.

## 2. Case study: thermal perception

We have a sensory system—commonly called the thermoreceptive system—that involves a series of distinct receptor populations in the skin (Schepers and Ringkamp, 2010). There are several different kinds of receptors involved, including thinly myelinated Aδ afferents that have receptive fields tuned to cooling and unmeylinated C afferents that code for both warming and cooling^1^. These various receptor populations systematically combine with other cutaneous systems (like those that code for pressure, vibration, and shape) to inform us about thermal properties in the distal environment (Jones and Lederman, 2006; Lumpkin and Caterina, 2007). They thus seem to be a crucial component of haptic touch (Fulkerson, 2014b). They also play an important role in our bodily awareness and the regulation of body temperature (Hammel and Pierce, 2002; Jones and Lederman, 2006), and so seem also to belong to our general systems of bodily awareness (which includes proprioception, kinesthesis, and our body schema). And finally, thermoreceptors also play an important role in the nociceptive system, informing us of bodily damage caused by extreme hot and cold stimuli^2^.

How should we classify this thermoreceptor system? Is it even one thing, given its many different afferent populations with distinct receptive fields and activation profiles? Maybe thermoreception itself is multisensory? We can also ask whether it is a part of touch. Should it be examined and investigated along with the other constituents of haptic awareness? Or are these thermoreceptors really part of the nociceptive system? Exposure to extreme heat and cold are, after all, among our most intense causes of pain. Then again, perhaps it is part of our general system for bodily awareness, since such thermoreceptors play such an important role in the regulation of a comfortable bodily state. In each of these cases, we can ask whether that makes touch, pain, and bodily awareness essentially multisensory ^3^.

**Figure 1:**
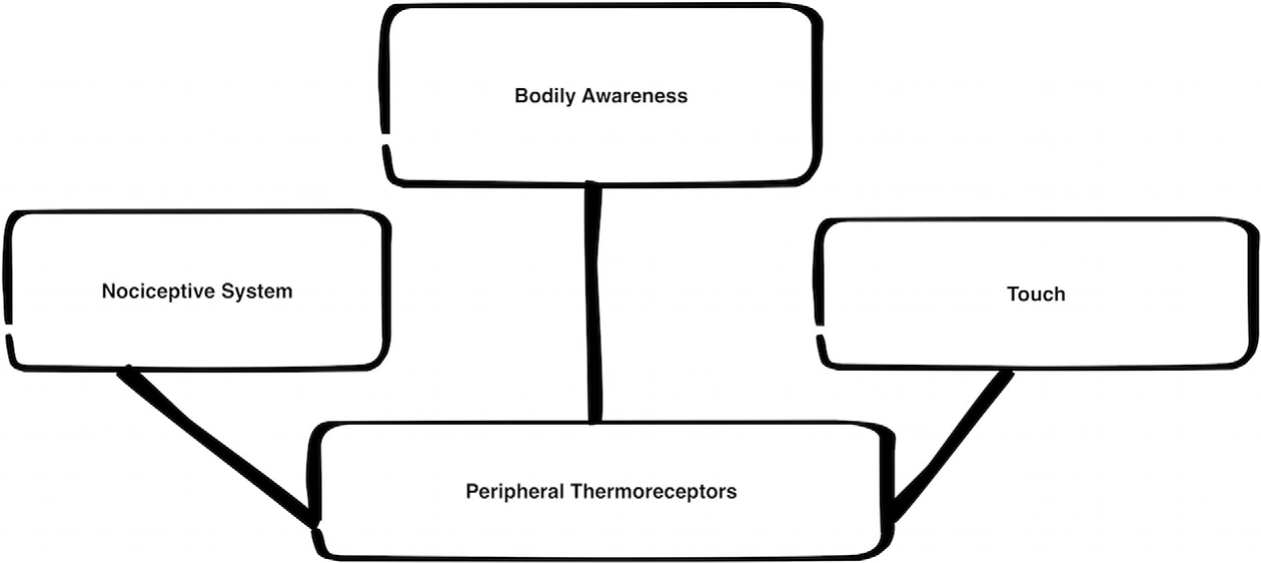
**Three ways of classifying thermoreceptors**.

Similarly, we might wonder whether thermoreception is its own independent sensory modality (multisensory or not). Is it *perceptual*, or do we only become informed of distal thermal properties indirectly, through inference from our bodily thermal state?^4^ Each of these positions has been defended (sometimes tacitly) in the literature (Martin, 1992; Schepers and Ringkamp, 2010; Gray, 2012). We are unlikely to make much progress on these claims, I believe, until we realize that there really is no such thing as the thermoreceptive system. The starting assumption that there is such a single system leads, I shall argue, to insurmountable theoretical and practical difficulties. Instead of a single thermalreceptive system, I believe that we have a complex series of receptors and processing units that perform *multiple* overlapping functions, and thus there are many, equally good ways of categorizing these various systems (see Figure 1). On this view, relative to one schema (its role in detecting and co-assigning features to distal objects), the thermoreceptive system is indeed continuous with (and therefore an essential part of) the sense of touch (itself a context-sensitive construct). If we focus purely on the physiological features of thermoreception, on the other hand, we have strong reason to classify (some elements of) this system as continuous with other elements of the nociceptive system. Like those other systems, many thermal channels involve slow, unmyelinated afferent nerve fibers that project contralaterally in the spinal column (unlike discriminatory touch afferents, which are typically myelinated and project ipsilaterally, Welsh, 2001). According to a third schema, we can see that thermoreceptors also play an important role in the awareness and regulation of body temperature, and can be classified as part of a larger system of bodily awareness that includes proprioception, vestibular awareness, and other regulatory systems (Wenger, 1995).

Let's focus on the details of this last claim, that our thermoreceptive system is part of a larger system of bodily awareness (the details here are useful as an illustration; this is not intended as an exhaustive argument about how to understand the thermal system). One useful way of categorizing sensory systems concerns whether they are outward-facing, giving us information about the external world, or body-facing, giving us information and performing functions primarily dedicated to bodily awareness and homeostatic self-maintenance. This distinction is often thought to be one between two separate systems: the *exteroceptive system* and the *interoceptive system*^5^. Exteroception, the story goes, provides us with information about the external world. It is largely informational and descriptive, giving us an evidence-like connection with the world around us. It is helpful for practical purposes (helping us find food and shelter and avoid dangers) but also for epistemic matters (helping us learn, form beliefs, and plan). Interoception, on the other hand, concerns the present state of our bodies. It is simply not in the business of directly reporting on what's going on in the external environment. Instead, this system is wholly concerned with *regulating* the present state of the body.

I want to focus on the defense of the view that thermoreception is interoceptive found in Craig (2002).

Craig suggests on the basis of physiological and functional connections that the thermal system should be categorized as part of the interoceptive system; that it tells us more about the present state of our bodies than it does about the external world (and that it does the latter only as a kind of secondary function). In doing so, it functions to maintain balance in our bodily system, and it does so in a way very similar to the operation of other homeostatic systems like those for hunger, thirst, and pain (see also Nakamura and Morrison, 2007). Here Craig (2002) describes the interoceptive system (emphasis mine):

This system is a homeostatic afferent pathway that conveys signals from small-diameter primary afferents that represent the physiological status of all tissues of the body. It projects first to autonomic and homeostatic centers in the spinal cord and brainstem, thereby providing the long-missing afferent complement of the efferent autonomic nervous system. Together with afferent activity that is relayed by the nucleus of the solitary tract (NTS), it generates a direct thalamocortical representation of the state of the body in primates that is crucial for temperature, pain, itch and other somatic feelings. This anatomical organization shows that these feelings are highly resolved, sensory aspects of ongoing homeostasis that represent the physiological condition of the body itself—*a distinct shift from the concept that pain and temperature are aspects of touch* (p. 655).

Notice that the key evidence for lumping these elements together into a single system are physiological. On my view, as we'll see, this is a perfectly appropriate context for categorizing these constituent systems. It just isn't the only such context.

While debates continue about whether hunger, thirst, and pain should be seen as perceptual (see e.g., Aydede, 2009), there is little debate about whether they represent something external to the body. On nearly all views, when we feel hungry, we are learning something about the present physiological state of our bodies rather than something about the external environment.

But why think that our thermoreceptive system (or rather many of its constituent parts) can play only one role, and must be either interoceptive or exteroceptive? A much better alternative here is to go pluralist: Craig is correct that there are explanatory schemes according to which it makes most sense to classify thermoreception along with other systems of bodily awareness like hunger and thirst. As Nakamura and Morrison (2007) write:

To evoke behavioral, autonomic, somatic and hormonal responses that counteract changes in environmental temperature before they affect body core temperature, thermoregulatory command neurons in the POA [the preoptic area] need to receive feedforward signaling of environmental temperature information from skin thermoreceptors through the spinal and trigeminal dorsal horns (p. 62).

Thermoreceptors, when they project to the POA and other areas that control homeostatic control, play a critical role in regulating our overall body temperature. We can recognize this without denying that we can *also* directly sense thermal features of the external environment when, for instance, our cutaneous thermoreceptors are co-activated with other constituents of externally directed haptic perception, whose afferents project directly to other areas of the somatosensory cortex.

Let's consider this second role in more detail^6^. When we actively explore an object with our hands, for instance, the synchronized motor engagement and cutaneous activation generate awareness of external objects and their thermal properties (Fulkerson, 2014b). This is, after all, how we successfully check whether the bath water is too hot, or whether the white wine is sufficiently chilled (cf. Jones and Lederman, 2006).

When we touch the bath water, we are attempting to determine the thermal state *of the water*. We reach our hand (or wrist, elbow, etc.) into the water, feel its temperature, and then decide whether or not the water is too hot. When we feel that the water is merely warm, we seem to succeed just fine in determining something about the state of the world, and not simply through some kind of explicit inference (Schepers and Ringkamp, 2010). Our experience is, it seems, about the state of the water. I've already suggested a functional reason for this: the activated thermoreceptors are not acting or interpreted on their own; they are temporally and spatially aligned with our exploratory actions and other cutaneous afferents in a way that unifies and enriches their informational content. This point can be supplemented by the fact that thermal properties bind to other external properties, forming complex tangible blends that involve the association of distinct distal properties. Thermal properties, for instance, turn out to be one of the essential elements in our experience of wetness (Sullivan, 1923; Ackerley et al., 2012) and material composition (Jones and Lederman, 2006), allowing us to differentiate an equally smooth wooden surface from a metal one. As Ackerley et al. (2012, p. 73) note: “Skin afferents are rarely composed of just one sensory modality and some sensory receptors are polymodal. Furthermore, perception of the sensation usually occurs from a blend of inputs, for example, when we sense that something is wet, it is typically due to changes in both touch and temperature afferents. There is no evidence to suggest that we have wetness receptors in the skin.” While these researchers often speak of touch and temperature awareness as separate things, their own evidence suggests otherwise. Relative to a purely physiological criteria, we can categorize touch and temperature as separate modalities, but when they function reliably to bring awareness of wetness and material composition they are better categorized as part of a single haptic system^7^. This is because material composition is one of the most important elements in tactual object-recognition (Klatzky and Lederman, 2008). None of these would be the case if thermal reception *only* informed us of the state of our bodies, or if external awareness involved a separate inferential step beyond the sensory level. Indeed, this issue is not unique to thermal awareness, since touch itself involves a great variety of distinct receptor types, projection sites, and downstream behavioral and psychological effects. As McGlone et al. (2007) end their discussion:

In conclusion, a dual role for touch serving both a discriminative and an affective role in human behavior has been described. The human hand has clearly evolved to perform a wide range of exploratory and manipulative tasks, and far surpasses this function in any other primate (p. 181).

So we can see that the thermoceptive system really does play (at least) dual roles in creatures like us. In addition, of course, the use of physiological and functional criteria to categorize the various constituents of the interoceptive system brings together a diverse range of systems that cross-cut in other interesting ways. While Craig suggests that all of these interoceptive systems have affective valence, for instance, so too do most externally-directed perceptual systems. Seeing something gruesome or disgusting often brings a strong emotional reaction. Vision and audition also play an important role in proprioception and wayfinding (Campos et al., 2012). While several of these systems also have some homeostatic function, they also differ in many other respects. The idea that there is a single “interoceptive system” is itself a useful explanatory context. We should not at all be surprised that there will be equally useful alternative ways of categorizing these systems.

Contrast Craig's view with that of Akins (1996). Like Craig, she denies that the senses are always in the business of veridically reporting on conditions external to an organism^8^.

She describes the traditional naturalistic account of senses, one that she will go on to deny, as follows: “The senses show the brain, otherwise blind, how things stand “out there,” both in the external world and in its own distal body” (p. 342). Later, she adds: “On the traditional picture, then, the senses, using a system of signals that capture the structure of a domain of external properties, tell the brain, without exaggeration or omission, “what is where” (p. 344).

This traditional (though still prevalent) view can motivate the idea that the senses have a single, hard-wired role to play (i.e., reporting external conditions directly to the brain). Conveniently for my purposes, she argues against this view with a detailed consideration of the peripheral thermoreceptive system. On the traditional view, “The receptors … must react with a unique signal, one that correlates with a particular temperature state.” (p. 342). Of course, this is not how peripheral thermoreceptors function. They have highly context sensitive and variable response rates that depend on the present state of the skin and embedded receptors, the context of activation, and the homeostatic needs of the organism (consider as Akins does the contrasting experiences generated by placing a warmed and a chilled hand in a neutral glass of water).

These facts lead Akins to suggest that sensory systems are “narcissistic”: while they sometimes convey information about the external environment, they always do so in a way that reflects first and foremost the needs and priorities of the organism. These needs, in turn, are often variable and highly context-sensitive. The senses involve many interacting parts, playing many different and important roles, but always *for the organism*. This perspective supports Craig's insights about the homeostatic and internally-directed nature of interoceptive thermal responses. Interoceptive contents, after all, will almost by definition be narcissistic. However, once the peripheral transducers are seen correctly as the initial components of much larger downstream neural networks subserving a variety of distinct psychological and behavioral activities, we can more easily see how the several channels involved in thermoreception can, in different contexts, and when connected with different downstream systems, be (literally) a part of several distinct sensory systems^9^.

The upshot then is not that Craig is wrong to apply the interoceptive category to some sensory systems. It's that he can be correct that there is an interesting and important way of connecting these systems, without excluding alternative ways of categorizing them. On the moderate pluralist view I will go on to defend, we can allow that thermal perception plays multiple different roles. Indeed, we can think of this system as a single system only by applying such a scheme of classification. There are a variety of distinct overlapping systems involved in thermoreception, and there are thus many different ways of classifying them. There is, on my view, both an internal and an externally-directed role. Thermoreception *really is* an important part of touch. It *really is* a part of our pain system. It *really is* part of bodily awareness. Which aspect we focus on depends on which aspects of the system we're interested in, and our explanatory purposes.

One thing is clear: even if one of the key functions of thermal perception is to provide information about the present state of our bodies, it does not follow that this is the *only* thing that thermal perception does. Or at least, it does not follow that there aren't multiple variants of thermal systems, all making use of the very same initial populations of peripheral thermoreceptors. One provides bodily information, another is connected with our haptic exploratory system, another plays a critical role in our pain experiences. Given this possibility, which I take to be an actuality, one should not make any inferences about perception generally on the basis of one function of thermal experience.

The upshot for us is that Craig highlights only *one* of the key functions of the thermal system, and his work allows us to see how (parts of) the same system can serve a variety of distinct roles. Some forms of thermal awareness only deliver awareness of the present state of our bodies; others inform us of the thermal properties of objects in our immediate environment. The real nature of thermoreception depends on what we are trying to explain, and on which associated features of the systems we are categorizing.

Thermoreception represents a kind of ideal case study here: it is a complex system, but well enough understood that we can use it to see exactly how plausible and powerful the moderate pluralist view can be. Now I will fill in the details of the sort of view I have in mind, starting with some essential background.

## 3. The importance of multisensory interaction

Recall the statements that began this paper: (i) We have many senses; (ii) They often interact.

These two statements were for a long time discounted by those in the cognitive sciences. Many had what O'Callaghan (2007, 2008) has called a “visuocentric” conception of perceptual experience. Visual experience was discussed to the exclusion of other modalities, and it was tacitly assumed that the conclusions reached for visual experiences would translate smoothly over to the other senses.

Recent work in cognitive science has accepted a more nuanced, multisensory conception of perceptual experience^10^. Empirically informed philosophy of mind has similarly seen a transformation in our understanding of perception^11^. Recent philosophy has seen a increase in research on other modalities^12^, sensory interactions^13^, and on the individuation of the senses^14^.

Of course, much of this philosophical work has been informed by and is directly responding to work in the various cognitive sciences exploring the deep interconnections and interactions between the senses.

Researchers have focused extensively on many different elements of sensory interaction, from cross-modal illusions, in which activations in one modality alter or suppress activations in another, to other categories of interaction like sensory facilitation, dominance, and several distinct forms of sensory integration. An increasing focus recently has been on more complex instances of sensory interaction like those occurring in affective experience, cognitive penetration, and synesthesia^15^. In all cases, researchers have extensively documented deep and pervasive interactions between sensory modalities.

These discoveries have largely undercut the “visuocentric” assumptions found in earlier research, and challenge many simplistic conceptions of sensory experience. Of course, one can still find work devoted entirely to vision (and to other individual modalities), but now such work is typically much more self-conscious about the limitations of focusing on a single modality studied in isolation. This recent shift has brought with it many important advances in our understanding of sensory interactions and the nature of perceptual consciousness, and has been a good thing for those of us trying to better understand the nature of perception.

As with any large shift in the scientific landscape, the new multisensory focus has also raised a number of important theoretical questions and posed novel challenges. I want to suggest that the move from our prior conception of separate individual senses requires more than merely investigating non-visual modalities or considering some sensory interactions. The move to a multisensory framework requires a more substantial reorientation of the theoretical landscape and of our investigative practices. At the same time, we should resist the urge to completely abandon all talk of senses and sensory systems. Instead, I will argue for an intermediate view that rejects any single, unified account of sensory modalities and their interactions, instead embracing a multitude of such accounts.

Before discussing these details, it's necessary to make two caveats. First, my focus in this paper is on the cognitive science classification of sensory systems. When I talk about *vision* or *audition*, I'm primarily interested in how we individuate and classify for the purposes of scientific explanation a particular part of our psychological biology. I am interested in the systems on the plausible assumption that it is those systems that are the constitutive and computational basis of the experiences generated^16^.

While this is a substantive assumption, the pluralist view does not depend on it (see the discussion of sensory substitution in §7.4 where this commitment is eased). It is an important advantage of my view that it allows and indeed embraces the idea that our perceptual experiences can be investigated and understood in multiple ways. So while the discussion that follows focuses almost exclusively on the sensory systems underlying our perceptual experiences rather than on their phenomenological, dynamic, or epistemic features, the view is ultimately sympathetic to many seemingly different approaches to understanding perceptual experience^17^.

Second, pluralist views have been discussed in a range of areas, especially in philosophy of biology (Kitcher, 1984; Mishler and Brandon, 1987; Ereshefsky, 1992; Steel, 2004; Cleland, 2013), but also in cognitive science (Dale et al., 2009), in general philosophy of science (Cartwright, 1999; Mitchell, 2002), aesthetics (Mag Uidhir and Magnus, 2011), and elsewhere. The view I defend in what follows was not initially inspired by this general move toward pluralism. Instead, it arose as a specific reaction to recent work on sensory interactions. It is not, therefore, the application of a form of pluralism defended in another domain to the sensory case. Instead, the view is motivated entirely by considerations internal to issues of explaining sensory interaction. For this reason, in what follows I will not engage in any systematic examination or comparisons between sensory pluralism and the many similar views defended in other domains, nor do I claim any special affiliation with such views.

## 4. Theoretical options

In this section, I want to spell out in general terms the nature of the tension forced on us by the move to a multisensory conception of perception, and consider the theoretical options.

We start with assumption (i) that we have many senses. An implicit assumption here is that these senses are more or less self-contained entities (it doesn't matter whether we think of them at this point as systems, modes of awareness, or forms of experience, etc.). One monist view that has been very influential is the claim that the senses are *modular input systems* (Fodor, 1983; Pylyshyn, 2006). On this view, the senses are domain specific, informationally encapsulated, hard-wired, and fast systems that function to process incoming sensory information. According to the modular account, we have a strong physiological, informational, functional, and computational distinction between sensory modalities. Vision uses different biological hardware than audition, to carry different information, for different computational and behavioral purposes^18^. The modular account is just one influential monist accounts in the literature. Its strength is supplemented by our strong intuitive sense that the senses represent very different forms of conscious awareness. What could be more clear than the difference between seeing something and hearing something? The view, and other weaker versions of monism, are systematically unable to adjust to the known facts about sensory interactions. This brings us to our second beginning statement.

That our senses interact (ii) seriously undermines any monist conception of sensory modality and interaction. We have learned that the senses interact in many interesting ways, often completely hidden from introspection. It has taken careful investigation to realize just how pervasive these influences can be. Much has been made, rightly, about the existence of cross-modal illusions (O'Callaghan, 2008). The McGurk effect shows that very often, what we hear is determined by what we see McGurk and MacDonald (1976); Skipper et al. (2007). The motion-bounce illusion shows that what we see is often party determined by what we hear (Sekuler et al., 1997). The use of brain scans and single-recording techniques has shown that many distinct areas of sensory cortex are active and engaged in the generation of experiences in single modalities (Ghazanfar and Schroeder, 2006). Similarly, vestibular and proprioceptive information influences activations in other modalities (Frissen et al., 2011; Campos et al., 2012). Motor movements influence cutaneous activations (Chapman, 1994). Thermal receptors influence pressure awareness (Jones and Lederman, 2006). What we see influences what we smell (Herz and von Clef, 2001). And on and on.

Once we realize just how pervasive and varied these interactions can be, we really start to lose grip on our what these separate senses involved are supposed to be. If they are not domain-specific, if they are not physiologically and informationally isolated, if they serve many varied and interactive functions, if the experiences they generate are fused into complexes that aren't easily decomposed or isolated in experience, then in what sense are they really distinct sensory systems *at all*? They certainly aren't isolated or independent. The facts of sensory interaction make it a very difficult theoretical challenge to say exactly what the senses referred to in (i) might actually be. Depending on how we think about multisensory interactions, it can become difficult to avoid the conclusion that we really don't have separate senses after all. Vision becomes a complex of various subsystems, each connected in various ways with many other sensory subsystems and aspects of cognition. Instead, we just have a vast mess of sensory interactions (maybe at the lowest level of sensory subsystems)^19^. I am not the first to notice these challenges. Consider the recent paper by Deroy et al. (2014), where they lay out many of the challenges facing the move to a multisensory conception of perception. As they note, there seem to be at present no clear experimental methods to directly investigate multisensory awareness or to distinguish between various models of sensory interaction. This problem is compounded, I believe, by appeal to several distinct forms of sensory interaction, including distinct levels of investigation (at the neurophysiological, behavioral, and introspective levels) and different forms of interaction (cross-modal influences, sensory blends, multimodal conjunctions). They are asking the right questions:

Can we simply take the current theories and protocols used to try and understand unisensory cases and then import them into the field of multisensory research? This is the approach that we wish to question here … shifting to multisensory cases is not cost-free for the study of perceptual awareness. It introduces both methodological and theoretical pressures. (Deroy et al., 2014, p. 3).

These pressures are compounded by the diversity of theoretical questions and experimental methods involved in these investigations. As they note later, “The recycling of unisensory protocols is unlikely to provide good ways to study multisensory awareness, if there is indeed such a thing” (Deroy et al., 2014, p. 8). My proposal suggests that these difficulties are not simply temporary impediments in our understanding of sensory awareness; they are the inevitable result of trying to fit a heterogeneous class of interactions under a single category (either unisensory vs multisensory, full stop). Consider a simplified example to support this claim.

Suppose that we are thinking about sensory interactions as occurring fundamentally between informational systems, and we characterize these (roughly) in terms of informational processing. If we do this, we can think about the interactions of the senses as constituted by the sharing and interaction among separate informational channels (for details on how this might go, see my 2011). What happens immediately, however, is that vision and audition no longer constitute anything like a single coherent sensory modality. They are complex systems that themselves involve interactions among disparate sensory subsystems sharing information in lots of interesting ways. *This happens for any other criteria we try to use to define sensory modalities* ^20^.

Since the interactions that operate in vision cross all kinds of boundaries, it becomes difficult to make sense of what counts as *the* visual system. Do those auditory processing centers that function reliably and consistently to contribute to the nature of our visual experiences count as part of vision? What about the pervasive influence of vestibular and proprioceptive systems on vision? And what do we mean by *visual experiences*? Once you start taking seriously the fact that the senses interact, and you start looking at the details of these interactions, it can be incredibly difficult to make sense of what we're actually talking about. The very idea of a *visual* system, or of a *visual* experience, starts to break down. So the worries we encountered with thermoreception are not unique to that domain; they are pervasive issues that arise for all putative perceptual modalities^21^.

As I see it, there are three ways to settle respond to this tension. We can preserve and supplement the status quo through some form of sensory monism, we can reject the entire project of sensory classification and go eliminativist, or we can go pluralist ^22^.

### 4.1. Option one

We can reject the claim that there is any tension or threat to the notion of a sensory modality posed by pervasive sensory interactions. One could, for instance, maintain the notion of the individual senses and try to explain multisensory interactions in ways that don't challenge the orthodox view of the senses. Connolly (2014) makes such an argument. In my commentary on Connolly's paper (Fulkerson, 2014a), I called such a view *sensory conservatism*; however, in this context I would describe it as a form of *sensory monism*. The idea is that we find some unified account that preserves the traditional notion of separate sensory modalities. Part of what this means is that we account for the wide range of sensory interactions by appeal to a criteria of sensory interaction that is independent from our criteria for being a sensory modality. We find a way to show that the traditional five (or more) senses remain of explanatory importance, and we account for multisensory interactions in a way that doesn't undermine these very kinds.

This is not an easy thing to pull off. For one, no one has yet suggested a criterion of sensory individuation that preserves the notion in the light of pervasive sensory interactions. The senses, whatever they are, cannot be domain specific, or functionally-unified, or marked in phenomenology, or physiologically specified, since what we call vision and audition and touch and olfaction and gustation have none of these features^23^.

### 4.2. Option two

Instead of sensory monism, one could opt for eliminativism. One could hold that the traditional senses (and their various interactions) are a kind of false construct or simplified idealization, and propose that we reject all such talk from our theorizing. Recent advances have demonstrated that our experience of the world is generated by a large number of interacting processing units. The natural way of thinking about sensory systems, on this view, is at a much finer grain than anything like modalities. Modalities are huge, messy collections of complex systems that involve mutually-interacting connections with numerous areas of the brain. They aren't natural kinds at all. On this view, to take the idea of sensory interactions seriously requires a much more radical shift in our thinking than we might have originally expected. In fact, it seems to require a rejection of (i). That is, it seems we ought to reject our intuitive notion of separate sensory modalities, and understand sensory interactions as pervasive “all the way down.” In the end, there really are no senses. This view has been defended most explicitly by Shimojo and Shams (2001), and one can see echoes of it in the work of many others (e.g., Driver and Spence, 2000; O'Callaghan, 2008).

### 4.3. Option three

Instead of adopting monism or eliminativism, I argue instead that we should adopt sensory pluralism. This is the view that there are indeed separate modalities, and natural ways of carving up sensory systems and their interactions, just like the monist believes; but like the eliminativist, the pluralist holds that no *single* account of modality and interaction is forthcoming. Against these views, the pluralist holds that there are *many* criteria of sensory interaction and unity, and these criteria in turn partly depend on our explanatory purposes and the investigative context. In other words, we should be pluralists about the senses and their interactions.

While some versions of pluralism can involve a radical ontology, the moderate view I have in mind is neither radical nor ontologically profligate. It simply holds that sensory systems are complexes that can be fruitfully engaged in many ways. Instead of calling it *pluralism*, one could, following Evans (1982) on reference, simply catalog and describe the *variety* of sensory interactions. Or, like Matthen (2010), one could focus on the *diversity* of sensory classification. These differences in terms do not track a real difference in the view I have in mind. I simply use the label *sensory pluralism* to name the view that sensory interactions come in many different forms, and therefore do not form a (single) natural kind.

My brand of sensory pluralism is moderate and constrained by the fact that there are indeed better and worse ways of dividing sensory interactions (more strongly: there are legitimate and illegitimate forms of sensory classification). Yet among the good ways, there are multiple equally useful options relative to our purposes. These cross-classify our perceptual systems in ways that can seem deeply at odds with each other, though in reality they enrich and mutually support our understanding of sensory experience. For instance, we can investigate as a single entity the causal-detection system composed of several seemingly distinct sensory modalities (Michotte, 1963). There is a legitimate theoretical and empirical question about whether this system of classification really is legitimate (as far as know, the jury is still out on this question). The pluralism I defend is thus modest rather than revisionary: it acknowledges the inherent complexity and deep interconnections between sensory systems at different processing levels, yet maintains that within this complexity there can be multiple robust explanatory systems of classification. We can, for instance, acknowledge in one explanatory context that all perception is inherently multisensory, while genuinely allowing in other contexts that some of our experiences are unisensory. My view is that what counts as a good classification of sensory interaction *partly* depends on the explanatory context. Hence “moderate sensory pluralism.”

In the next section, I want to highlight those general features of pluralist systems that ground legitimate schemes of classification, and support their explanatory utility.

## 5. The case for pluralism

I put forward here some general claims in defense of the kind of moderate pluralism I have mind. This will necessarily be a simplified discussion concerning various explanatory strategies we might take with respect to a domain. There are many discussions of pluralism in the literature, and the basic tenants are well understood. As Mitchell (2002, p. 55) remarks, “The “fact” of pluralism in science is no surprise. On scanning contemporary journals, books, and conference topics in some sciences, one is struck by the multiplicity of models, theoretical approaches, and explanations.” This seems especially true of cognitive science (Dale, 2008; Dale et al., 2009). And, I shall argue, it is also the way we ought to be thinking about sensory interactions.

**Figure 2:**
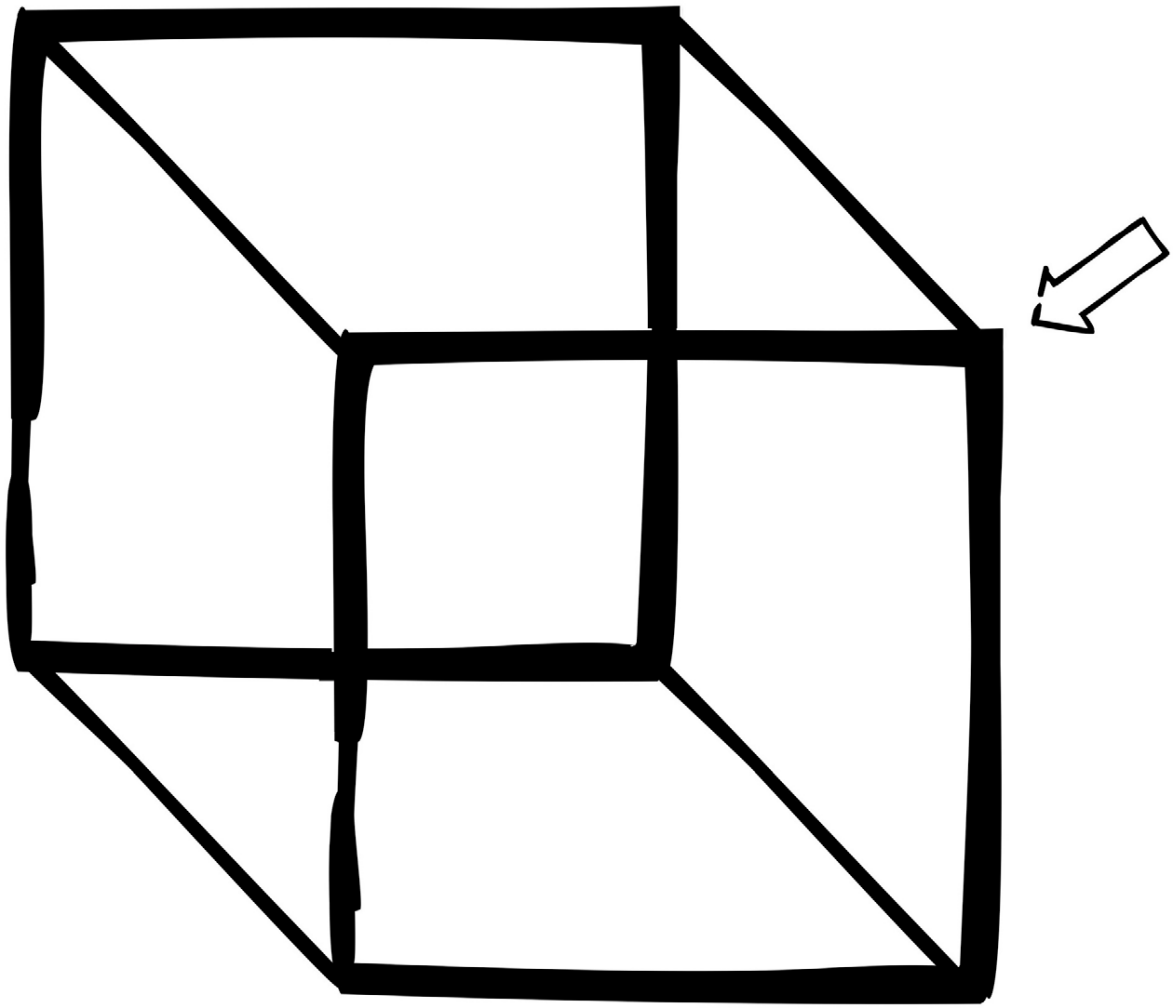
**Necker cube**.

There are some general formal features that any complex system subject to moderate pluralism should exhibit. These are *decomposition*, *functional overlap*, and *bounded recombination*. We can find analogs of these features in something as simple as a Necker cube (see Figure 2).

The Necker Cube is a basic, simplified model of the kind of pluralist view I have in mind: it has constituent parts (decomposition). These parts in turn equally satisfy two inconsistent high-level descriptions, and they do so because any particular part (a line or intersection) can play more than one role (functional overlap). There are also limits to the shapes that the cube can take on (bounded recombination); while there are multiple ways of seeing the cube, these ways are highly constrained^24^.

Look at the point in the upper corner of the image picked out by the arrow. According to one high-level description, the point is a *front-facing* top corner of a cube. According to the other, it is a *rear-facing* point on the bottom corner of the cube. Which is correct? Well, the natural answer is *both*, depending on how the cube is seen. The parts themselves don't settle the answer since they are consistent with both views. The lines and points on the page satisfy two distinct high-level descriptions. These are highly constrained descriptions: there are only two of them for these points, and they are very precise. In fact, fixing the high-level description completely fixes the role played by these elements. In context, there are correct and incorrect descriptions of these features. Many options for this point are completely ruled out: this particular point cannot be a rear-facing top corner. Nor can it stop being a point, and so on.

In this very basic example we can see the features of a moderate form of pluralism, one without problematic metaphysical commitments. Let us take these simple lessons and formalize them a bit for proper application to the sensory domain.

### 5.1. Decomposition

Let's start with decomposition. It is not enough that a system has parts, but that it has *functionally salient* parts. This means that the system must have some *functional* decomposition. I am being intentionally broad about my use of “function” here. The parts of sensory systems I'm interested in play different causal, informational, mechanical, computational, and structural roles. As might be suspected from this list, I'm not here interested in, and nothing in my view hangs on, defending a particular theoretical account of *function*^25^. The only constraint is that the relevant notion of function not be sensitive to our interpretive or conventional uses. There must be some actual intrinsic basis on which the low-level functions are assigned^26^. I have in mind something like a “natural” or “proper” function, though very broadly construed (for a discussion of function in this sense, see Dretske, 1991 and Millikan, 1989).

A system is functionally decompositional—in the broad sense of function alluded to above—just in case its operation can be broken down into simpler parts that operate separately from the other constituent parts^27^. In other words, the complex system needs to be composed of parts that have a specifiable functional identity. A simple feature, like a point in space, or a complex object with lots of parts that each play no distinctive functional role, does not admit of the kind of moderate pluralism I have in mind. A hunk of iron for instance, is not subject to *this* sort of pluralism. It is surely a natural kind, one that can be used to do lots of different things^28^. There are versions of pluralism that would apply to hunks of iron (Havstad, 2014), but for my purposes it doesn't count because its parts and their functions are too simple to admit of multiple appropriate schemes of scientific classification.

This should not be surprising: work in the metaphysics of mind has long recognized that psychological systems require a minimal level of functional complexity^29^. Some entities simply do not have the structure or complexity necessary to allow for equally robust categorization at high levels^30^.

Such decomposition is a necessary condition, but not a sufficient one. Just because something can be broken down into functional parts, it does not follow that we should be pluralists about its high-level nature. Another feature is needed, and that is functional overlap.

### 5.2. Functional overlap

A pluralist friendly system must have a minimal level of complexity. In addition, however, the relationship between the constituent parts is also important. In particular, those functional parts should each contribute to distinct high-level systems.

It's unlikely that a system that had single-function parts (or more accurately, parts that contributed only to a single more complex system) could generate any interesting form of pluralism. This is simply because there would be only a single role played by that part, and so only a limited number of ways to reconceive its role in the larger system. Think about the cutting wheel on a can opener. It is an essential constituent part of the opener that plays a specialized functional role. So we have decomposition. But there's only the one role for the cutting wheel to play. It doesn't serve any other purpose, or contribute in different ways to different complexes at higher levels of the can-opener. It's therefore difficult to suggest that our understanding and classification of the can opener varies in any way with the explanatory context^31^. Typically speaking, it doesn't. Only when the parts start to take on multiple roles will we start to see interesting ways of combining them into higher level systems.

Think again about the toy example of the Necker cube. Each point on the page functions both as a facing side and a rear side, depending on the view taken. That single point on the page (or the screen) plays *both* roles. If there were not some functional overlap, then there would not be multiple perspectives available on the cube as a whole. The parts serve dual functions, and we are able to see them play these different roles in distinct high-level structures. What role it plays thus depends on which high-level structure we're interested in (more on this soon).

### 5.3. Limited recombination

The final feature is limited recombination. We can think of this as an upper bound on our pluralism. There are many ways of being a sensory system, on my view. And so our theoretical accounts of such systems are open to several distinct systems of classification. And yet, despite the existence of multiple forms of sensory interaction and methods of classification, these are highly constrained. There are clear limits on the roles played by the constituent parts and on the larger systems in which they participate. The view is not “anything goes.” This is what makes this form of pluralism *moderate*. There are clear objective constraints that ground the admissible conceptions of the constituent systems^32^.

While one can look at the marks of the Necker cube on the page and see distinct but legitimate shapes represented, one cannot find spheres or other shapes in the mix. The objective locations of the points and lines rule out most shape interpretations. So there are clear constraints on how we understand this relatively simple system. Not only does this make the view ontologically moderate, it is also what makes the multiple views explanatory. The claim is not that sensory systems can be understood however we like, or that there are not facts of the matter concerning the natures of sensory interactions. Instead, the idea is that there are multiple, objectively robust roles played by the constituent elements of sensory systems, and so for any particular constituent subsystem, there will be more than one role it plays in distinct higher-level systems, but not an unlimited number of such roles. As we've seen, this perfectly describes the peripheral thermoreceptor system. This system contributes to pain awareness and is thus part of the nociceptive system. It also contributes to object recognition and externally-directed thermal awareness, and so it is also an essential part of the sense of touch. It also plays an important role (along with central thermoreceptors) in the regulation of body temperature, and so is part of our homeostatic regulatory system (along with thirst and hunger). There is no single classification of these peripheral thermoreceptor populations, because they play many varied roles in our lives.

We can now see that certain complex systems are amenable to a modest pluralist view. To suggest that a single complex system can be understood and classified in multiple ways does not commit us to a problematic ontology. We can make this clear by making explicit the contextual operator in our sensory classification. A claim such as “System X is multisensory” leaves out this operator, and thus cannot be properly evaluated. It is not an explanatory statement. Instead, we should be evaluating claims of the form “System X is multisensory according to explanatory schema Y.” This schema specifies the respect in which something counts as multisensory or not. Similarly for other claims.

Adding this sort of clause will allow researchers to avoid mere terminological disputes and help clarify the nature of the investigation in question. One worry about pluralist views is that they can foster confusion and hinder scientific progress. I find compelling the reply in Ereshefsky (1992, p. 680) to such worries about pluralistic views of species in biology:

[B]iologists should categorize those lineages by the criteria used to segment them: interbreeding units, monophyletic units, and ecological units. The term “species” is superfluous beyond the reference to a segmentation criterion; and when the term is used alone it leads to confusion. The term “species” has out-lived its usefulness and should be replaced by terms that more accurately describe the different types of lineages that biologists refer to as “species.”

Similarly, philosophers and others talking about sensory experience should avoid using terms like “multisensory,” “multimodal,”or “cross-modal” without being clear about the way in which they are using those terms. They should not assume that there is a single, theoretically interesting way in which senses interact, or that we can have, say, a single unified account of what qualifies as a “cross-modal” form of interaction. Some interactions are legitimately unisensory, others involve activations of processing units distributed widely in other systems (and these often overlap!). There is thus no single way for these systems to interact; they are complexes that interact in many theoretically interesting ways.

Putting all of these elements together, we can see that sensory systems should be ideally situated to the kind of pluralistic view I've been outlining. They are, after all, evolved biological systems that serve many functions, and are subject to many constraints. Indeed, all of cognition seems amendable to this perspective. As Dale et al. (2009) write: “The mind, as somehow constituted by brainbodyenvironment interaction, is extraordinarily complex. In addition, we have many and assorted interests in that interaction” (p. 1). And these parts play these roles in a number of ways, through informational extraction and computation, through behavioral and bodily features and reactions, and so on.

## 6. Sensory systems

It should be clear from the gloss above that sensory systems are ideal candidates to satisfy all three constraints. If we've learned anything over the last few decades, it's that our sensory systems are deeply complex structures that involve a large number of interacting elements. Very often these elements are put to different uses by various downstream systems. As such, sensory systems are decomposable into functionally salient parts. These parts (rods, cones, retinal ganglion cells, etc.) in turn perform different functions depending on which downstream systems they are contributing to^33^. And so it should not be surprising that the explanatory context—the kinds of systems we're investigating and what behaviors and capacities we seek to explain—can have a significant impact on how the various systems are categorized and understood.

Even entirely within vision this should be clear. A cone cell examined in isolation performs one function (converting electromagnetic energy into neural signals), but the function it serves can be influenced by, and in turn influence, other cones connected to it (for instance, when detecting edges). At higher levels of complexity, these same constituent elements can perform many other functions. For instance, these early visual elements are essential parts of a complex object-recognition system, but also play a role in guiding our motor actions. Recent debates about the “two visual streams hypothesis” arise partly because of these dual roles (Milner and Goodale, 1995). Which stream is *really* vision? Various options are available here, but taking the pluralist conception, one can see that we shouldn't expect a single answer. What we call “vision” is in reality a complex set of distinct systems and subsystems. There are *many* things that count as vision (this is the pluralism). Which one is going to be explanatory and relevant for scientific purposes depends on making clear the explanatory context. Even so, it does not depend *entirely* on the context; there are clear objective constraints limiting the ways we can think about visual experiences. The view is heavily grounded in the actual capacities and functions of the constituent elements of the system.

While it strengthens the claims I'll be making that they mesh with actual practice in the cognitive sciences, the case for sensory pluralism doesn't rest *entirely* on this descriptive enterprise. It is neither necessary nor sufficient for the truth of sensory pluralism that researchers engage in these strategies of classification (they could simply be mistaken in their current practices). My purpose also is similarly not to weigh in on or take sides on these first-order debates. Instead, the discussion is meant to show how fruitful, plausible, and powerful the pluralist perspective can be in helping further our understanding of difficult issues in recent work on perceptual experience. Moderate sensory pluralism is, ideally, a form of what Mitchell (2002) calls “compatible pluralism.” On this view, the various explanations involved are not strict competitors, but mutually supporting accounts of complex phenomena:

[C]omplex phenomenon harbor multiple interacting causal processes and multiple levels of organization which all may be involved in the generation of the feature to be explained. By disambiguating the question to be answered by an explanation–i.e., what is the evolutionary origin of a trait or behavior we observe now—one is still left with a plurality of potential causes acting at a number of levels of organization which may well constitute compatible answers to that single question (Mitchell, 2002, p. 57)

We have seen how this perspective enriches our understanding of the thermoreceptive system. Let us see how it might apply to other recent debates in the literature^34^.

## 7. Implications

I will now briefly discuss some potential applications of the account described here in several domains of active research on sensory awareness.

### 7.1. Olfaction

The olfactory system is another obvious case where sensory pluralism finds ample support. Intuitively, we believe that we have a single “sense of smell” and that we can understand the components of this system as a single, coherent thing. The reality is a bit more complicated. We in fact have two senses of smell, a orthonasal and a retronasal system. The orthonasal system involves molecules that are picked up in the surrounding environment through the nasal cavity, often by exploratory acts of sniffing (Wilson and Stevenson, 2006). These inputs provide reliable information about the nature of environmental chemical stimulants (See also Batty, 2009). We can even use smell for wayfinding and to help influence our emotional reactions (Herz, 2007; Rosenblum, 2011).

Retronasal olfaction by contrast involves chemical irritants that rise from inside the mouth and pass through the olfactory epithelium from the other direction. Though the initial activation sites are more or less the same in both instances, the resulting perceptual experiences and functional interactions are very different from orthonasal ones. Here the smell becomes fused and combined with other taste information and generates a complex experience of flavor (Auvray and Spence, 2008). So is smell a unified sensory modality? Is it externally directed? Or is it part of a multisensory system of flavor detection? According to the sensory pluralist, the answer is all of the above. The initial chemoreceptors involved in both systems might be the same, but they play very different roles when combined with distinct inputs (external vs internal sources of chemical irritants) and co-processing elements (sniffing and head movements in externally directed tasks and coordinated taste and texture activations in the mouth, respectively). Here again we see that what seems like a single modality is really a complex collection of interacting elements that can be appropriately classified in a variety of ways.

### 7.2. Auditory processors

The auditory system also admits of several distinct schemes of classification: the initial processing units involved in auditory experience play a role in several interacting systems: general sound perception, our awareness of speech, and in the perception of music. There are reasons for thinking of these as very different systems, and thus there are multiple ways of classifying and accounting for our auditory awareness; these ways involve different functional roles, associated interactions with other systems, and behavioral capacities. In addition, of course, we can understand audition as part of larger networks connected to causal detection (as in the motion bounce illusion, Sekuler et al., 1997). All of these forms of classification are robust and explanatory, and often involve the same initial processing units and transducer populations. We shouldn't expect a single, unified account of audition. Like thermoreception and smell, it involves a range of capacities that admit of distinct forms of classification.

### 7.3. Synesthesia

Synesthesia is another interesting case for understanding sensory pluralism. This condition involves (roughly) the reliable activation of one modality by stimuli presented to another. In this way, it seems to be a kind of cross-modal interaction, but one importantly different from typical cases of multisensory integration or facilitation. In addition, it poses a number of basic definitional and phenomenological questions. Researchers have long known, for instance, that synesthesia comes in a variety of forms, and it is difficult to find a single account that covers all (and only) genuine cases (Macpherson, 2007; Mroczko-Wasowicz and Werning, 2012)^35^. Given the difficulties in presenting a robust, unified account of synesthesia, we should not be surprised if it turns out that there are multiple forms of the condition, each distinctive in various ways. The pluralist perspective suggests that we should not (simply) hold out for a single mechanism underlying the overall condition, but explore the possibility that the condition arises in a variety of distinctive ways. One could even allow that so-called normal subjects might exhibit features continuous with the possession of synesthesia (see Auvray and Deroy, in press; Cohen, in press). The question of what counts and what doesn't count as synesthesia in general might not be a well-formed question. Maybe synesthesia isn't a natural kind at all?^36^

The sensory pluralist can allow that, in some respects, many cases of synesthesia are extensions of ordinary perceptual capacities, part of the same functional units that underlie our general experience of the world. On the other hand, from a slightly different explanatory context, we can see discontinuities as well. In addition, some forms of the condition might be more strongly connected with one context, and might exploit resources typical in ordinary perceptual interaction, whereas others might involve interactions more difficult to reconcile with typical sensory interactions. There is no reason to choose sides here (at least not yet); once we make clear the explanatory context of our investigations, and the precise nature of the interactions under investigation, we can make clear the sense in which these phenomena are like and unlike other forms of sensory awareness. What this involves, as in the other cases I've discussed, is making clear the explanatory context and embracing the idea that there may be multiple useful ways of investigating and theorizing about these interactions^37^.

### 7.4. Sensory substitution

There is one final area of intensive investigation that would benefit from the pluralist perspective. Sensory substitution and enhancement devices pose many challenges for traditional monist accounts of sensory individuation. Such devices provide input usually provided by one modality through a device that interacts with a different modality. For instance, a camera might be used to provide inputs to touch for a subject without normal sight. If a subject is presented with visual information through a camera system that translated those signals into a vibrating plate on the tongue, does the resulting experience count as visual or tactual? There have been many discussions about such devices^38^.

The pluralist view suggests that these devices ought to admit of distinct forms of classification. They pose such a difficulty because they often have characteristics from both modalities. If we focus on behavioral capacities we might classify the experiences generated by the device one way; if we focus instead on phenomenal character we might classify it differently. Enhancement systems might produce novel forms of awareness that don't fit into any of our current schemes of classification. They also suggest that the focus of this discussion—the multiple roles that our low level biological machinery can play—might be too narrow. Sensory enhancement and substitution might reveal that our sensory capacities outstrip the present functions of our hardware^39^.

## 8. Summing up

The main alternative view to pluralism would be some form of monism: the idea that a single scheme of classification should define each of the sensory modalities, and their interactions. But it should be clear from the preceding that it seems highly unlikely that we will find a unified criteria for defining each of the senses. Vision differs from the other senses in a multitude of ways, and plays many distinct roles at different levels of sensory processing. What single account of modality or interaction can capture that diversity, and then work equally well for audition, proprioception, touch, taste, vestibular awareness, sensory dominance, facilitation, suppression, and cross-modal blends (like flavor)?

Others might worry that I've left the details here are a little spare. That is intentional. I do not wish to commit myself to any particular account of scientific explanation here. If one takes a mechanistic or functional explanation as ideal for work in cognitive science, then what I say here suggests that we can (and should) focus on a diversity of functional explanations when it comes to the senses and their interactions. If one prefers a different explanatory framework (a computational or informational story, say), then my claims here should motivate us to look for a diversity of computational processes involved in the generation of sensory experience.

Nothing that I've said requires us to take a stand on intertheoretic relations, reductionism, emergence, or explanation. At no point do I claim that there are sensory systems that can or cannot be reduced to lower level functional or computational components. The claim is that, when it comes to sensory systems, we should expect distinct explanatory accounts to be available (cf. Dale et al., 2009). The only substantive commitment I make is that each system of classification be genuinely explanatory, and grounded in the objective basic features of the system. In this sense, it is a genuine ontological pluralism (cf. Ereshefsky, 1992), but a moderate one. My claim is not that we cannot know what senses “really are.” It is that, as a matter of fact, senses really are lots of things, and what counts as explanatory in our theorizing about sensory interactions depends on how we're carving the systems up and what we are trying to explain. So while my point is distinct from claims about multiple realizability and about levels of explanation in the cognitive sciences (see Marr, 1982; Dennett, 1989), the view is both compatible with and offered in the spirit of these views.

As we've seen, there has been a lot of work recently on understanding the nature of multisensory awareness. Arguments abound concerning whether we need to completely reject our prior conceptions of sensory modalities and their interactions, or whether we can salvage some aspects of sensory unity and cohesion. The sensory pluralist view doesn't, in itself, settle these debates. But it does suggest that many of these debates are merely verbal disputes, where the contexts of investigation and explanation have not been clarified. There need be no debate, for instance, between those who think of thermoreception as continuous with pain and other interoceptive systems, and those who investigate the role of thermoreception in object recognition and sensory exploration. There should be no disputes between views on which flavor awareness forms a separate modality or not. In some explanatory contexts it most certainly does; in others it need not. The pluralist view doesn't give up on the idea of correct scientific theorizing, it just makes clear something that already is the case: sensory systems and their interactions are complex, multifaceted, and occur at many levels of processing. Our theorizing about these interactions needs to recognize and take on board these complexities^40^.

### Conflict of interest statement

The author declares that the research was conducted in the absence of any commercial or financial relationships that could be construed as a potential conflict of interest.

## Footnotes

^1^As we'll see, the different response patterns of these many afferent channels play different roles in different contexts. For instance, the thinly myelinated Aδ fibers play an especially important role in our perception of wetness (Ackerley et al., 2012).

^2^That pain you feel when eating spicy foods? It's caused by the activation of thermoreceptors. Black pepper contains *piperine* and chili peppers contain *capsaicin*, both of which both activate TPRV1 thermoreceptors. See Caterina et al. (1997).

^3^Nociception represents another ideal case study in the difficulties facing any unified account of sensory interactions. For a convincing argument here see Corns (2014). For earlier discussion see Aydede and Guzeldere (2002).

^4^See for example Gray (2012) for a nuanced discussion of what our thermal experiences might represent even if focussed on the body.

^5^It is interesting to compare this with the distinction between *emotional* and *discriminative* touch (McGlone et al., 2007). These categories overlap in several respects; indeed, McGlone et al. (2007) use some of Craig's findings and terminology to help mark the difference between emotional and discriminative. However, they note some interesting cases of overlap between physiologically distinct afferent populations (p. 176), and, as we'll see, they end up defending a dualist (i.e., pluralist) view of touch.

^6^I set aside for now the details concerning the role of thermoreception in nociception.

^7^Indeed, I would argue that our tendency to talk of “thermoreception” and “touch” as unified entities also involves eliding important physiological and functional differences between the large number of distinct receptor populations involved in each system. We'll return to this general tendency in a later section.

^8^Her focus is on sensory *contents* rather than processing, but the general point is the same.

^9^I am grateful here to Kathleen Akins for extremely helpful discussion of this material.

^10^The literature here is enormous; a good place to start is Stein (2012).

^11^The transition in philosophy was largely spearheaded by O'Callaghan (2008, 2007). Evans (1982) and Martin (1992) are other important early sources. For the most recent treatment, see the articles in Bennett and Hill (2014).

^12^See for instance, recent work on smell (Batty, 2010; Richardson, 2011); on taste (Smith, 2009, 2012); on sounds (Nudds, 2001; Matthen, 2010), and on touch (O'Shaughnessy, 1989; Scott, 2001; Ratcliffe, 2008; Richardson, 2013; Fulkerson, 2014b). In addition, there has been work on many forms of experience outside the traditional five senses, for instance on temporal experience (Grush, 2005; Phillips, 2008; Lee, 2014) and on bodily awareness (de Vignemont, 2007; Schwenkler, 2011).

^13^O'Callaghan (2012); Macpherson (2011); Bennett and Hill (2014)

^14^See for example Keeley (2002); Gray (2005); Nudds (2003); Macpherson (2010); Matthen (in press).

^15^See for example Mroczko-Wasowicz and Nikolic (2014); Vuilleumier and Driver (2007); Stokes (2012); Siegel (2011); Villemure et al. (2003).

^16^While my own preference is for representational and computational approaches to the mind, this assumption is not intended to rule out approaches to cognition and perception that emphasize the strong connections between this biology and the external world. For instance, it should not be taken to exclude views like those recently defended by Noë (2004); Hurley (1998); Thompson (2007). Indeed, as I say in the text, it is an advantage of my position that it leaves space for a variety of explanatory approaches to the study of perceptual experience, including embodied and enactive views. For example, my view leaves space for contexts in which a view of modality along the lines defended by McGann (2010) is appropriate. (A critical difference is that McGann's view is strongly eliminativist, holding that “there is no such thing as an experience that is purely visual, auditory, or otherwise modal” (p. 72). By my lights, whether there are such experiences is dependent on the explanatory context, and what we mean by “visual,” “auditory,” and the like.)

^17^Though I should emphasize that, in my own view, such approaches are useless unless constrained and informed by the empirical facts.

^18^Keeley (2002) can be read in some ways as regimenting many of these criteria as a means of individuating sensory modalities. In my view it is the strongest statement of sensory monism, but as it predates the current interest in multisensory perception, it does not even make an attempt to incorporate facts about sensory interactions.

^19^A view like this seems to be advocated by Shimojo and Shams (2001).

^20^See Fulkerson (2011) for one elaboration of this claim.

^21^I want to emphasize that, while I speak here and throughout about the classification of sensory *modalities*, my target is broader than issues about the individuation of the senses. My primary concern, in fact, concerns how best to classify and understand lower level interactions between the senses.

^22^This list is not exhaustive; there could be various forms of hybrid view in the area. I have difficulty imagining any hybrid view that was not consistent with the moderate pluralism I'm advocating here.

^23^I have defended the idea that our best account of the individual modalities is that they are collections of sensory subsystems that function together to group or bind sets of sensory features together (Fulkerson, 2014a,b). This claim was made in the context of sensory pluralism: this is just *one way* to categorize the subsystems that make up vision. In many other respects, vision really is multisensory. On my view, it really is both, depending on what framework of investigation we are using.

^24^I'm using the Necker cube here as a basic model to point out those features of sensory systems that make them amenable to a pluralist view. Obviously, the analogy is not perfect. In particular, the cube lacks the required functional complexity, and its competing views are not sensitive to the *explanatory* context. Still, I think it is a useful toy example for clarifying the view I actually have in mind. I am grateful to an anonymous referee for pressing me to clarify this point.

^25^There is, of course, a very large literature on the nature of function and mechanism in the special sciences (especially cognitive science and biology). For a very brief introduction, see Bechtel (2008); Cummins (1985); Machamer et al. (2000); Feest (2003); Millikan (1989)

^26^Consider again the Necker cube. While it's high level description depends on the visual context, the status of the lines and points on the page do not depend on the context. Each cube can be broken down into distinct functional complexes (corners and sides and edges, etc.).

^27^In Fulkerson (2011) I discuss a version of this view, which I called the *functional dissociation criterion*. The notion I'm using here is broader than what I had in mind previously.

^28^Perhaps this is a good place to note that what I have in mind differs significantly from the notion of *multiple realizability* in the cognitive sciences. The idea here isn't that the “same high level description” can be realized by different underlying constituents as in multiple realization, but that the very same set of underlying constituents can be parts of equally salient, but distinct high-level systems. My view is thus something closer to the converse of multiple realizability.

^29^See for instance, see the discussion in Block (1997) concerning the “Disney Principle,” the idea that in the real world, anything with a mind needs to have a minimal level of complexity (unlike the sentient teapots and spoons in the world of cartoons). These debates arise in several domains, involving debates about reduction, emergence, and the relationship between low-level realizers and high-level descriptions (Batterman, 2000).

^30^It is of course my view, given the above, that there are several distinct notions of pluralism and that we need to be careful not to assume that they are equivalent.

^31^Of course, this is a bit simplified. Maybe for some explanatory purposes the material composition of the cutting wheel matters (why did it rust?), for others it might be its size or shape (why won't it cut this can?). These points suggest that pluralism is a robust phenomenon throughout our explanatory practices. The present point is that, above and beyond these basic forms of explanatory pluralism, sensory systems exhibit an additional layer of complexity.

^32^Compare the *sorting* and *motivating* principles discussed by Ereshefsky (1992).

^33^The notion of “downstream” systems can be deeply misleading. Sensory systems, like much of cognition, is deeply heterarchical, and involves processing going in multiple directions at the same time. These complications only add additional support to the claims made here.

^34^The pluralist view offers a robust explanation for the prevalence of such debates. More importantly, it suggests a way to move forward on such debates, as I hope will become clear in each example.

^35^See also Simner (2012a) target paper, commentaries by Eagleman (2012) and Cohen Kadosh and Terhune (2012), with a reply by Simner (2012b).

^36^Gray (2001) makes the case that synesthesia poses problems for Fodorian modularity, and should be taken seriously in any account of psychological kinds. As one might expect, I agree with this assessment, and suggest in addition that facts about synesthesia also support a version of sensory pluralism.

^37^I should emphasize, one final time, that nothing I've said here is meant to rule out genuine disagreements; there can and will be false accounts of the phenomena that can be definitely ruled out even if sensory pluralism is true.

^38^For a representative discussion, see (among others): Deroy and Auvray (2014); Noë (2004); Auvray and Myin (2009); Deroy (2012); Farina (2013); Rita and Kercel (2003); Froese et al. (2012).

^39^See Clark (2003) for discussion along these lines.

^40^I would like to thank the editor and referees for an extremeley helpful set of comments on earlier versions of this paper. I would also like to thank the audience and participants at the Network for Sensory Research Workshop on Multisensory Perception held at the University of Toronto where an early version of this material was presented.
